# Factors Associated with Infant Feeding Methods after the Nuclear Power Plant Accident in Fukushima: Data from the Pregnancy and Birth Survey for the Fiscal Year 2011 Fukushima Health Management Survey

**DOI:** 10.1007/s10995-016-1973-5

**Published:** 2016-03-30

**Authors:** Kayoko Ishii, Aya Goto, Misao Ota, Seiji Yasumura, Masafumi Abe, Keiya Fujimori, Pregnancy and Birth Survey Group of the Fukushima Health Management Survey

**Affiliations:** Radiation Medical Science Center for the Fukushima Health Management Survey, Fukushima Medical University, 1 Hikarigaoka, Fukushima City, Fukushima Prefecture 960-1295 Japan; Department of Public Health, Fukushima Medical University, Fukushima, Japan; Department of Midwifery and Maternal Nursing, Fukushima Medical University, Fukushima, Japan; Department of Obstetrics and Gynecology, Fukushima Medical University, Fukushima, Japan

**Keywords:** Formula feeding, Breastfeeding, Infant feeding methods, Disaster, Fukushima nuclear accident

## Abstract

*Objectives* The objective of this study was to assess the frequency of and factors associated with infant feeding methods after the Fukushima nuclear power plant accident using data from the Fukushima Health Management Survey. *Methods* We conducted an anonymous self-administered questionnaire survey of 16,001 women who gave birth around the time of the Great East Japan Earthquake and registered their pregnancies at Fukushima Prefecture municipal offices between August 1, 2010 and July 31, 2011. The responses of 8366 women were analyzed. Chi square tests and multiple logistic regression analysis were used to compare various factors between women who had formula-fed their children because of concern regarding radioactive contamination or other reasons and those who had breastfed exclusively. *Results* The percentage of women who had breastfed exclusively was 30.9 %. The percentage of women who had both breastfed and formula-fed or formula-fed exclusively was 69.1 %, of which 20.3 % formula-fed because of concern regarding radioactive contamination of breast milk. The use of formula feeding because of concern about radioactive contamination was significantly higher in women who had resided within the evacuation area and those whose regular antenatal care had been interrupted. The use of formula feeding for other reasons was significantly higher in women who had resided within the evacuation area and lower for those who had willingly switched to another medical institution. *Conclusions for Practice* Our results suggest the importance of providing breastfeeding support to women who are forced to evacuate or whose antenatal care is interrupted after a disaster.

## Significance

Many previous studies have identified factors associated with infant feeding methods. However, few studies have investigated the factors, including concern regarding radioactive contamination of breast milk, that influence infant feeding methods after a nuclear accident. Our study revealed that the use of formula feeding was associated with residence in the disaster-affected area and interruption of antenatal care. Our findings highlight an importance of breast feeding support after the nuclear accident.

## Introduction

Breastfeeding is the best infant feeding method and helps to establish the bond between mother and child (Japanese Ministry of Health, Labour, and Welfare [MHLW] [13]. The United Nations Children’s Fund (UNICEF) and World Health Organization both promote breastfeeding and jointly published the “Ten Steps to Successful Breastfeeding” in 1989. The State of The World’s Children 2015 published by the UNICEF reported that the worldwide average rate of exclusive breastfeeding within 6 months was 38 % [23]. In Japan, the MHLW has set a target goal of 60 % for infants being breastfed at 1 month after birth as part of its “Healthy Parents and Children 21” campaign. Although the MHLW’s 2005 National Nutrition Survey on Preschool Children found that 96 % of pregnant women were hoping to breastfeed, the breastfeeding rate after childbirth was only 51.6 % at 1–2 months and 55.8 % at 4–5 months even when temporal formula feeding (e.g., while going out) was defined as breastfeeding [14]. Factors associated with the discontinuation of breastfeeding include prenatal and postnatal depression, attachment disorder, smoking status, low birth weight, perceptions of insufficient breast milk supply, and inappropriate hospital support [3, 10, 16, 26]. On the other hand, factors that have been shown to be associated with the continuation of breastfeeding include high self-efficacy and husband or partner support [10, 17].

A government-funded survey was established in 2011 to respond to concerns regarding the possible radioactive contamination of breast milk after the accident at the Tokyo Electric Power Company’s Fukushima Daiichi Nuclear Power Plant caused by the Great East Japan Earthquake on March 11, 2011. Based on the results of this survey, six associated organizations issued a statement stating that continuation of breastfeeding had no negative effect on children’s health [18]. Fukushima Medical University also launched the Fukushima Health Management Survey in the fiscal year (FY) 2011, which was commissioned by the Fukushima Prefectural Government. [25]. The survey results showed almost no difference in the rates of complications, such as stillbirth, miscarriage, abortion, premature birth, low birth weight, and congenital deformities, between the affected areas and national data [5].

In this study, we analyzed the frequency of and factors associated with infant feeding methods after the Fukushima nuclear power plant accident using data from the FY2011 Fukushima Health Management Survey (the first year this survey was carried out). Specifically, we examined formula feeding-related factors by classifying the subjects into the following two groups: mothers who used formula feeding because of concern about the influence of radiation exposure and mothers who used formula feeding because of other reasons. The disaster-related factors of interest were the residential region, continuation of scheduled antenatal care, and switching to another medical institution of their own accord. The overall aim of the study was to assess the need for breastfeeding support after a disaster.

## Methods

### Subjects and Eligibility Criteria

The subjects of this study were 16,001 women who registered their pregnancies at municipal offices within or outside the Fukushima Prefecture between August 1, 2010 and July 31, 2011 and who underwent antenatal examination or gave birth on or after March 11, 2011. Self-administered questionnaires were mailed by post, and responses were received from 9321 of the 16,001 women (58.3 % response rate). Invalid responses and responses from women who had given birth before the disaster, resided outside the prefecture, had twins, or had suffered miscarriage or stillbirth were excluded. Thus, the responses from 8366 women were analyzed. This study was approved by the Ethical Review Committee of Fukushima Medical University (13051). The study objective was described on the cover of the questionnaire, and the return of the questionnaire was regarded as consent.

### Definitions of Variables

- *Feeding method* The possible responses to the question “What infant feeding method have you used to date (until the start of weaning)?” were “Breastfeeding only,” “Combination of formula feeding and breastfeeding,” and “Formula feeding only.”
- *Reason for formula feeding* Respondents who answered “Combination of formula feeding and breast-feeding” and “Formula feeding only” were asked the question “What was your reason for formula feeding?” Possible responses included “Insufficient breast milk,” “Concern about the effect of radiation exposure on breast milk,” and “Other reason.”
- *Evacuation area* Respondents who had registered their pregnancies with any of the following 13 municipal governments were defined as residents of the evacuation area: Tamura, Minamisoma, Kawamata, Hirono, Naraha, Tomioka, Kawauchi, Okuma, Futaba, Namie, Katsurao, Iitate, and Date. The evacuation area was in the coastal region, and was characterized by a lower mean income per person and a lower ratio of students going on to college as compared to the respective mean values for the prefecture [7].
- *Switched to another facility either within or outside the Fukushima Prefecture of their own accord* Respondents who answered the question “Did you continue to receive antenatal care and give birth at the medical institution where you were originally scheduled to undergo this care?” with the responses, “Switched to a different institution within Fukushima Prefecture of my own accord” or “Switched to a different institution in another prefecture of my own accord,” were included in this group.
- *Depressive symptoms* Respondents who answered “Yes” to at least one of the following two questions were regarded as having depressive symptoms: “During the past month, have you often felt low or depressed?” and “During the past month, have you often felt that you cannot become interested in anything or that you cannot enjoy things wholeheartedly?”

### Statistical Methods

Statistical analyses were performed using IBM SPSS Statistics 21.0. Respondents who had used either formula feeding exclusively or a combination of breastfeeding and formula feeding were divided into two groups based on their reason for formula feeding, i.e., concern regarding radioactive contamination and other reasons (e.g. lack of breast milk). Various factors were compared between these two groups and the group that breastfed exclusively. Univariate analysis using the Chi square test was first carried out for the following factors: disaster-related factors (residential area, continuation of scheduled antenatal care, switched to another facility either within or outside the Fukushima Prefecture of their own accord), maternal factors (age, parity, depressive symptoms), obstetric factors (type of pregnancy, illness during pregnancy, delivery method), and child-related factors (sex of child, birth weight, congenital anomaly, neonatal asphyxia). Multiple logistic regression analysis was then used to investigate the association between disaster-related factors and formula feeding after adjusting for significant factors in the univariate analysis.

## Results

### Subject Characteristics

Subject characteristics are shown in Table 1. The mean subject age, mean gestational age, and mean birth weight were 30.1 ± 5.0 years, 39.0 ± 1.6 weeks, and 3030 ± 402 g, respectively. The mean elapsed time from delivery to questionnaire completion (baby age) and mean duration between disaster and delivery were 24.5 ± 13.6 weeks, and 23.4 ± 13.8 weeks, respectively. In addition, the minimum, 25th percentile, median, 75th percentile, and maximum values for subject age (15, 27, 30, 34, and 51, respectively), mean gestational age (22, 38, 39, 40, and 44, respectively), mean birth weight (402, 2788, 3034, 3280, and 4935, respectively), elapsed time from delivery to questionnaire completion (0, 14, 24, 35, and 92, respectively), and duration between disaster and delivery (0, 12, 23, 34, and 56, respectively) were determined.

**Table 1:** Subject characteristics (*n* = 8366)

Item^a^	Mean ± SD^b^	*n* (%)
Maternal age (years)	30.1 ± 5.0	
Elapsed time from delivery to questionnaire completion (weeks)	24.5 ± 13.6	
Gestational age (weeks)	39.0 ± 1.6	
Birth weight (g)	3030 ± 402	
Duration between disaster and delivery (weeks)	23.4 ± 13.8	
Feeding method before weaning		
Combination of breastfeeding and formula feeding		5187 (62.2)
Exclusive breastfeeding		2575 (30.9)
Exclusive formula feeding		578 (6.9)

^a^Missing values have been omitted for each item

^b^ *SD* standard deviation

### Frequency of and Reasons for Formula Feeding

Of the 8366 women surveyed, 2575 (30.9 %) had breastfed exclusively and 5765 (69.1 %) had both breastfed and formula-fed or formula-fed exclusively (Table 1). Among the latter group, the most common reason for formula feeding was insufficient breast milk (4166/5689; 73.2 %), followed by concern regarding radioactive contamination of breast milk (1154/5689; 20.3 %) and other reasons (1006/5689; 17.7 %).

### Type of Water Used for Formula Preparation

The most common type of water used for formula preparation was bottled water, accounting for 75.5 % of cases (*n* = 4295; Table 2). Tap water was used significantly more often by women who formula-fed for reasons other than concern about radioactive contamination (*p* < 0.001), and bottled water was used significantly more often by those who formula-fed because of concern about radioactive contamination (*p* < 0.001).

**Table 2:** Type of water used for formula preparation according to the reason for formula feeding

Type of water^a^	Total (*n* = 5689)	Formula-fed because of concern about radioactive contamination (*n* = 1154)	Formula-fed for other reasons (*n* = 4535)	*p* value
	*n* (%)	*n* (%)	*n* (%)	
Tap water				
Yes	1729 (30.4)	207 (17.9)	1522 (33.6)	<0.001
No	3960 (69.6)	947 (82.1)	3013 (66.4)	
Bottled water				
Yes	4295 (75.5)	1019 (88.3)	3276 (72.2)	<0.001
No	1394 (24.5)	135 (11.7)	1259 (27.8)	
Other (e.g., well water)				
Yes	178 (3.1)	30 (2.6)	148 (3.3)	0.25
No	5511 (96.9)	1124 (97.4)	4387 (96.7)	

^a^Missing values were excluded

### Comparison of Women Who Formula-Fed Because of Concern Regarding Radioactive Contamination and Those Who Breastfed Exclusively

Compared with women who breastfed exclusively (*n* = 2575), women who formula-fed because of concern regarding radioactive contamination (*n* = 1154) were significantly more likely to have the following characteristics (Table 3): resident of the evacuation area (*p* < 0.01), interrupted antenatal care (*p* < 0.001), depressive symptoms (*p* < 0.001), illness during pregnancy (*p* < 0.01), and cesarean section delivery (*p* < 0.001).

**Table 3:** Comparison of women who formula-fed because of concern about radioactive contamination or other reasons and women who breastfed exclusively

	Formula-fed because of concern about radioactive contamination (*n* = 1154)	Formula-fed for other reasons (*n* = 4535)	Breastfed exclusively (*n* = 2575)	Formula-fed because of concern about radioactive contamination versus breastfed exclusively	Formula-fed for other reasons versus breastfed exclusively
	*n* (%)	*n* (%)	*n* (%)	*p* value^a^	*p* value^a^
*Disaster-related factors*					
Residential region					
Within evacuation area	163 (14.1)	580 (12.8)	281 (10.9)	<0.01	0.02
Outside evacuation area	991 (85.9)	3955 (87.2)	2294 (89.1)		
Continuation of scheduled antenatal care					
Yes	845 (73.8)	3709 (82.4)	2077 (81.2)	<0.001	0.23
No	300 (26.2)	794 (17.6)	480 (18.8)		
Switched to different medical institution of own accord					
Yes	233 (20.2)	797 (17.6)	587 (22.8)	0.08	<0.001
No	921 (79.8)	3738 (82.4)	1988 (77.2)		
*Maternal factors*					
Age (years)					
<35	939 (81.4)	3557 (78.4)	2138 (83.0)	0.22	<0.001
≥35	215 (18.6)	978 (21.6)	437 (17.0)		
Parity					
Primipara	284 (24.6)	1340 (29.5)	654 (25.4)	0.61	<0.001
Multipara	870 (75.4)	3195 (70.5)	1921 (74.6)		
Depressive symptoms					
Yes	462 (40.5)	1147 (25.6)	647 (25.4)	<0.001	0.90
No	678 (59.5)	3336 (74.4)	1896 (74.6)		
*Obstetric factors*					
Duration between disaster and delivery (weeks)					
<23	705 (61.1)	2110 (46.6)	1235 (48.0)	<0.001	0.26
≥23	449 (38.9)	2422 (53.4)	1340 (52.0)		
Type of pregnancy					
Natural pregnancy	1110 (96.4)	4325 (95.5)	2473 (96.3)	0.79	0.13
Other types^b^	41 (3.6)	203 (4.5)	96 (3.7)		
Illness during pregnancy (either before or after the disaster)					
Yes	312 (27.0)	1048 (23.1)	570 (22.1)	<0.01	0.35
No	842 (73.0)	3487 (76.9)	2005 (77.9)		
Delivery method					
Vaginal delivery	887 (78.6)	3506 (78.5)	2130 (83.8)	<0.001	<0.001
Cesarean section	241 (21.4)	959 (21.5)	413 (16.2)		
*Child-related factors*					
Sex of child					
Male	598 (52.1)	2331 (51.8)	1310 (51.2)	0.60	0.64
Female	549 (47.9)	2171 (48.2)	1249 (48.8)		
Birth weight (g)					
<2500	81 (7.1)	376 (8.3)	154 (6.0)	0.22	<0.001
≥2500	1065 (92.9)	4130 (91.7)	2407 (94.0)		
Congenital anomaly					
Yes	25 (2.3)	130 (3.0)	56 (2.3)	1.00	0.08
No	1085 (97.7)	4250 (97.0)	2428 (97.7)		
Neonatal asphyxia					
Yes	14 (1.3)	53 (1.2)	18 (0.7)	0.12	0.05
No	1101 (98.7)	4316 (98.8)	2472 (99.3)		

^a^Chi square test was used

^b^Other types include ovarian hyperstimulation, artificial insemination, and in vitro fertilization

### Comparison of Women Who Formula-Fed for Reasons Other Than Concern Regarding Radioactive Contamination and Those Who Breastfed Exclusively

Compared with women who breastfed exclusively (*n* = 2575), women who formula-fed for reasons other than concern regarding radioactive contamination (*n* = 4535) were significantly more likely to be a resident of the evacuation area (*p* = 0.02), aged ≥ 35 years (*p* < 0.001), and primipara (*p* < 0.001). Moreover, the likelihood of formula feeding for those switching to another institution either within or outside the Fukushima Prefecture of their own accord was low (*p* < 0.001), but that for those having a cesarean section (*p* < 0.001) and a low-birth weight baby (*p* < 0.001) was high (Table 3).

### Association Between Disaster-Related Factors and Formula Feeding Because of Concern Regarding Radioactive Contamination

Residence in the evacuation area [odds ratio (OR) = 1.28] and interrupted antenatal care (OR = 1.48) were significantly associated with formula feeding because of concern regarding radioactive contamination (Table 4). The ORs for residence in the evacuation area (OR = 1.40) and interrupted antenatal care (OR = 1.96) were even greater in a subanalysis of women whose only reason for formula feeding was concern regarding radioactive contamination (*n* = 622).

**Table 4:** Association between disaster-related factors and formula feeding because of concern about radioactive contamination

Disaster-related factors	Univariate^a^		Multivariate^b^	
	OR^c^	95 % CI^d^	OR	95 % CI
Residential region				
Within evacuation area (vs outside evacuation area)	1.34	1.09–1.65	1.27	1.02–1.58
Continuation of scheduled antenatal care				
No (vs yes)	1.54	1.30–1.81	1.31	1.09–1.57
Switched to different medical institution of own accord				
Yes (vs no)	0.86	0.72–1.02		

^a^Independent variables were 1 = formula feeding because of concern about radioactive contamination and 0 = exclusive breast feeding

^b^Adjusted for maternal factors (age, parity, and depressive symptoms), obstetric factors (duration between disaster and delivery, type of pregnancy, illness during pregnancy, and delivery method), and child-related factors (gender, body weight, congenital anomalies, and neonatal asphyxia)

^c^ *CI* confidence interval

^d^ *OR* odds ratio

### Association Between Disaster-Related Factors and Formula Feeding for Reasons Other Than Radioactive Contamination

Residing in the evacuation area (OR = 1.20) and switching to a different medical institution (OR = 0.73) were associated with formula feeding for reasons other than radioactive contamination (Table 5).

**Table 5:** Association between disaster-related factors and formula feeding because of reasons other than concern regarding radioactive contamination

Disaster-related factors	Univariate^a^		Multivariate^b^	
	OR^c^	95 % CI^d^	OR	95 % CI
Residential region				
Within evacuation area (vs Outside evacuation area)	1.20	1.03–1.39	1.20	1.03–1.41
Continuation of scheduled antenatal care				
No (vs Yes)	0.93	0.82–1.05		
Switched to different medical institution of own accord				
Yes (vs No)	0.72	0.64–0.81	0.74	0.65–0.84

^a^Independent variables were 1 = formula feeding because of other reasons and 0 = exclusive breast feeding

^b^Adjusted for maternal factors (age, parity, and depressive symptoms), obstetric factors (duration between disaster and delivery, type of pregnancy, illness during pregnancy, and delivery method), and child-related factors (gender, body weight, congenital anomalies, and neonatal asphyxia)

^c^ *CI* confidence interval

^d^ *OR* odds ratio

## Discussion

### Need for Breastfeeding Support After the Disaster

The 2010 National Growth Survey on Preschool Children, a nationwide survey, found that the rate of exclusive breastfeeding was 50 % at all points between 1 and 5 months of age [12]. According to the 2000 National Growth Survey on Preschool Children, the breastfeeding rate after childbirth was 44.8 % at 1–2 months and 35.9 % at 4–5 months; the breastfeeding rate in Japan progressively increased in the 10 years thereafter [14]. Thus, the breastfeeding rate in our survey (30.9 %) was lower than the expected national figure. However, the breastfeeding rate in Fukushima Prefecture was reportedly lower than the national figure even before the disaster. In a 2007 survey by the Fukushima Bonyu no Kai (Fukushima Breastfeeding Association), the reported breastfeeding rate at age 4 months was 32.2 % [6].

From the survey data, two groups were created for analysis based on the duration between the disaster and the delivery. The group in which the period between the disaster and delivery was shorter showed a higher rate of formula feeding due to concern regarding the influence of radiation exposure on breast milk. Underlying the low breastfeeding rate in Fukushima were concerns regarding the transmission of radioactive substances to children through breast milk, which led mothers to formula-feed despite being able to breastfeed. The Infant and Child Feeding in Emergencies (IFE) Core Group coordinated by the Emergency Nutrition Network issued guidelines on “How to Ensure Appropriate Infant and Young Child Feeding in Emergencies” [9]. The IFE advised that the use of a nursing bottle and comforter should be actively discouraged in such disasters. Furthermore, the American Academy of Pediatrics (AAP) issued “Infant Feeding in Disasters and Emergencies” guidelines to provide guidance on radiation exposure [1]. The AAP reported that breastfeeding should not be stopped if an appropriate dose of iodine is administered at an appropriate time according to the advice of local health officials. Contamination of breast milk with iodine-131 was found even in women residing more than 200 km away from the Fukushima nuclear power plant [24]. However, according to the team that conducted the abovementioned government survey, the amounts of radioactive substances detected in the breast milk of seven women immediately after the disaster were extremely small, and continuation of breastfeeding would have no negative effect on their children’s health [18]. Opportunities must be created to educate pregnant women on the importance of breastfeeding and to motivate them to breastfeed [2]. In light of the nuclear accident, it is not only important to convey the advantages of breastfeeding but also to provide information on radioactivity in a user-friendly form to help women not to discontinue breastfeeding because of uncertainty over radioactive contamination. A clear national guideline is needed for nuclear disaster preparedness.

### Need for Breastfeeding Support for Women in the Evacuation Area

Our study results showed that more women who had resided in the evacuation area formula-fed than breastfed exclusively regardless of the reason for formula feeding. Stress affects the sympathetic nervous and adrenal systems, which diminish milk production and inhibit let-down [8]. Fatigue caused by evacuation, an unstable lifestyle in a new place, insufficient fluid intake, and poor breast milk secretion due to stress may have potentially contributed to the low breastfeeding rate. Soon after the disaster, an ample supply of formula was reportedly available. Some breastfeeding women supplemented breast milk with formula out of anxiety, while others chose to formula-feed because of hygiene-related concerns [19, 22]. In times of disaster, women may be more inclined to continue breastfeeding if carefully tailored support, in terms of providing adequate fluids, food, blankets, and other supplies, and ensuring privacy, is made available [4].

### Need for Mental Health Care from Pregnancy to Delivery

We found that a higher proportion of women whose antenatal care was interrupted formula-fed because of concerns regarding radioactive contamination. Previous studies have shown that a sense of coherence (the ability to counter stress for oneself) during pregnancy may be predictive of mental health during the puerperal period [20]. Furthermore, difficulties encountered during an unexpected event, such as a nuclear accident, may affect self-perception skills [11]. These studies suggest that particular attention should be paid to providing mental health care to women whose scheduled antenatal program is interrupted.

### The Importance of Empowerment

Contrary to antenatal care interruption, the breastfeeding rate was higher for those who switched to a different medical institution within or outside Fukushima Prefecture of their own accord. The higher breastfeeding rate among women who switched to a different medical institution may have been due to a greater sense of safety and self-efficacy. Previous studies have shown a high rate of exclusive and continued breastfeeding among women with high self-efficacy [15]. Increasing a women’s sense of self-efficacy and offering empowering advice may promote breastfeeding [2, 21]. Women whose medical care was interrupted may have been more vulnerable to the unexpected event and associated loss of control. Thus, efforts to provide such women with appropriate counseling and support to increase their sense of control should be undertaken.

## Limitations of the Study

There are four major limitations. First, the survey response rate was only 60 %. Therefore, the data as a whole may have been biased. Second, it was impossible to estimate the exact breastfeeding rate because of the definition of exclusive breastfeeding (breast milk only until weaning) and variations in the timing of the responses. Third, the socioeconomic status and the possibility of other potential confounders were not measured at a personal level. Fourth, in the evacuation area, the population was lower while the response rate was higher. Therefore, the area outside the evacuation area showed a higher healthy respondent bias than the evacuation area, which might have influenced the results.

## Summary

Before weaning, 30.9 % of women had breastfed exclusively and 69 % of women had both breastfed and formula-fed or formula-fed exclusively. Although insufficient breast milk (73.2 %) was the most common reason for formula feeding, 20.3 % of women responded that their reason for formula feeding was concern regarding the effect of radioactive contamination on breast milk. Bottled water was the most frequently used type of water for formula preparation and was used significantly more often by women who formula-fed because of concern regarding radioactive contamination. The OR for formula feeding because of concern about the effect of radioactive contamination on breast milk was significantly higher for women who had resided in the evacuation area and those whose scheduled antenatal care was interrupted. Women who had resided in the evacuation area were also significantly more likely to formula-feed for reasons other than concern about radioactive contamination. On the other hand, those who had switched to another medical facility within or outside the Fukushima Prefecture of their own accord were likely to continue breastfeeding.
